# The paediatric version of Wisconsin gait scale, adaptation for children with hemiplegic cerebral palsy: a prospective observational study

**DOI:** 10.1186/s12887-018-1273-x

**Published:** 2018-09-15

**Authors:** Agnieszka Guzik, Mariusz Drużbicki, Andrzej Kwolek, Grzegorz Przysada, Katarzyna Bazarnik-Mucha, Magdalena Szczepanik, Andżelina Wolan-Nieroda, Marek Sobolewski

**Affiliations:** 1 Institute of Physiotherapy, University of Rzeszów, Warszawska 26 a, 35-205 Rzeszów, Poland; 2 Rzeszów University of Technology, Rzeszów, Poland

**Keywords:** Hemiplegic gait, Cerebral palsy, Wisconsin gait scale, Intra-observer reliability, Inter-observer reliability, Scale adaptation

## Abstract

**Background:**

In clinical practice there is a need for a specific scale enabling detailed and multifactorial assessment of gait in children with spastic hemiplegic cerebral palsy. The practical value of the present study is linked with the attempts to find a new, affordable, easy-to-use tool for gait assessment in children with spastic hemiplegic cerebral palsy. The objective of the study is to evaluate the Wisconsin Gait Scale (WGS) in terms of its inter- and intra-rater reliability in observational assessment of walking in children with hemiplegic cerebral palsy.

**Methods:**

The study was conducted in a group of 34 patients with hemiplegic cerebral palsy. At the first stage, the original version of the ordinal WGS was used. The WGS, consisting of four subscales, evaluates fourteen gait parameters which can be observed during consecutive gait phases. At the second stage, a modification was introduced in the kinematics description of the knee and weight shift, in relation to the original scale*.* The same video recordings were rescored using the new, paediatric version of the WGS. Three independent examiners performed the assessment twice. Inter and intra-observer reliability of the modified WGS were determined.

**Results:**

The findings show very high inter- and intra-observer reliability of the modified WGS. This was reflected by a lack of systematically oriented differences between the repeated measurements, very high value of Spearman’s rank correlation coefficient 0.9 ≤ |R| < 1, very high value of ICC > 0.9, and low value of CV < 2.5% for the specific physical therapists.

**Conclusions:**

The new, ordinal, paediatric version of WGS, proposed by the authors, seems to be useful as an additional tool that can be used in qualitative observational gait assessment of children with spastic hemiplegic cerebral palsy. Practical dimension of the study lies in the fact that it proposes a simple, easy-to-use tool for a global gait assessment in children with spastic hemiplegic cerebral palsy. However, further research is needed to validate the modified WGS by comparing it to other observational scales and objective 3-dimensional spatiotemporal and kinematic gait parameters.

**Trial registration:**

anzctr.org.au, ID: ACTRN12617000436370. Registered 24 March 2017.

## Background

Development of children with cerebral palsy is determined by the degree of intellectual disability and the associated learning ability which mostly determines participation in society [1, 2]. In functional assessment, mobility is also important [3, 4]. In cerebral palsy gait pattern functions and walking can be impaired. Neuromusculoskeletal impairment may be related to muscle function and control of voluntary movement functions [5].

Walking analysis in children with cerebral palsy is a sensitive tool used in evaluating progress resulting from treatment, enabling accurate assessment of functional performance and providing information necessary for determining goals of therapy [6, 7]. Advanced methods of assessing gait in this group of patients enable in-depth multidimensional analysis, yet they require considerable financial resources and sophisticated non-standard equipment due to which they are often inaccessible. On the other hand, observational gait analysis, an affordable method which can be used easily and quickly, is commonly applied in the clinical practice as a basic tool for evaluating gait abnormalities in children with cerebral palsy [6–8]. In observational gait assessment the examiner performs visual analysis of gait pattern using video recordings and scales describing abnormalities in both temporospatial and kinematic parameters of gait [9]. In the literature there are few studies focusing on tools designed for assessment of children with spastic cerebral palsy, therefore their clinical use cannot be judged based on the existing evidence [6]. Scales enabling assessment of gait in children with cerebral palsy include: Observational Gait Scale [10], Visual Gait Assessment Scale [11], Salford Gait Tool [12], and Edinburgh Visual Gait Scale [13]. However, the first of the above scales is only used for documenting gait changes in children after injections of botulinum toxin A [10], otherwise it does not present good results for all evaluated parameters [7]; the second scale can achieve only reliable sagittal plane assessment of the knee and ankle, yet it is not a reliable tool for assessing sagittal plane hip motion and additionally, it does not attempt to characterise either transverse or coronal plane deviations [11]; similarly the third scale is only sagittal plane observational gait assessment tool [12]; finally, the last scale on the above list is most extensive and detailed, enabling analysis in other planes of motion, yet just like all the others it focuses exclusively on assessing kinematic gait parameters [13]. In the clinical practice there is a need for a simple and practicable tool enabling detailed and multifactorial gait assessment (i.e. taking into account all the planes as well as spatiotemporal and kinematic parameters) and monitoring of rehabilitation outcomes, specifically in children with spastic hemiplegic cerebral palsy.

According to many researchers the Wisconsin Gait Scale (WGS) is a valuable tool which can easily be used in observational analysis, enabling detailed and accurate multidimensional assessment of spatiotemporal and kinematic gait parameters and evaluation of progress achieved in gait re-education by patients with hemiplegia, yet it is designed for adult stroke patients [14–18]. However, gait in children with hemiplegic cerebral palsy is very similar to gait observed in adult individuals with hemiplegia after stroke. It is also characterised by decreased walking speed, longer stance phase and shorter swing phase on unaffected leg, longer gait cycle, short stride, high stride frequency, impaired motor coordination and stability during walking; additionally, there are significant differences in kinematic parameters of the hip, knee, and ankle joints compared to healthy children [19, 20]. This observation provided inspiration for the present study and for the attempt to adapt WGS for children with spastic hemiplegic cerebral palsy. Moreover, it has been suggested by some researchers that psychometric properties of WGS should be analysed in more detail in patients with various neurological disorders other than stroke [21]. The practical value of the present study is linked with the attempts to find a new, affordable, easy-to-use tool for gait assessment in children with spastic hemiplegic cerebral palsy. The main objective of the study is to assess WGS in terms of its inter- and intra-observer reliability in observational gait analysis based on examination of video recording of children with hemiplegic cerebral palsy.

## Methods

### Participants and setting

**Fig. 1:**
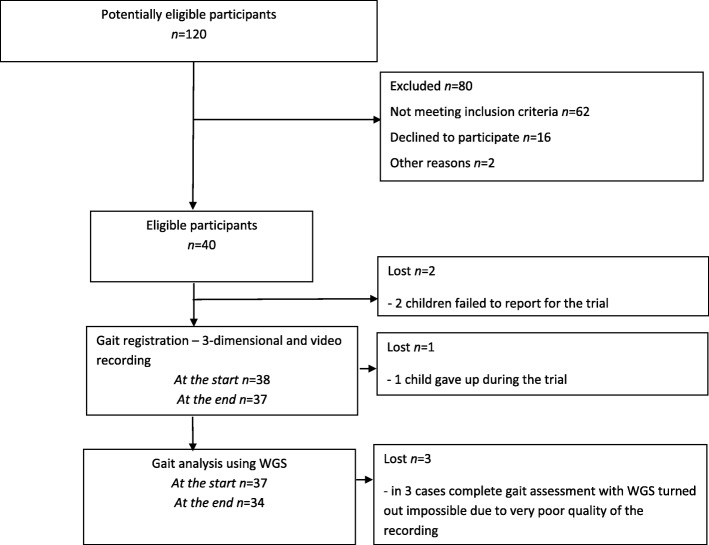
Flow of subjects through the study

The study was carried out in a group of 34 patients with hemiplegic cerebral palsy. It was conducted at University of Rzeszów gait laboratory. Inclusion criteria: hemiplegic cerebral palsy, age 6–18 years, independent gait without assistance of another person (with use of walking aids or AFO orthosis - if necessary). Exclusion criteria: cognitive function deficits impairing the ability to understand and follow instructions, unstable medical condition, differences in the length of extremities exceeding two centimetres, surgical intervention in the area of lower extremities less than 6 months before the study, and botulinum toxin treatment less than 6 months before the study. A total of 56 patients participating in outpatient rehabilitation program at the Regional Hospital No. 2 in Rzeszów in 2014–2016, who met the inclusion criteria, were selected out of 120 patients with a medical history of cerebral palsy. After being contacted by phone, 40 caregivers agreed for their children to participate in the gait analysis, however two children failed to report for the trial, one child gave up during the trial and in three cases complete gait assessment on WGS turned out impossible due to very poor quality of the recording. Finally, WGS based gait analysis was performed for 34 children. Figure 1 shows the flow of the subjects through the study and Table 1 presents the characteristics of the group.

**Table 1:** Baseline characteristics of individuals with cerebral palsy

	Group (*n* = 34)
Age [years], mean (sd)	10.9 (2.3)
Sex [female/male]	19/15
Paretic limb [right/left]	19/15
Height [cm], mean (sd)	138.9 (11.26)
Weight [kg], mean (sd)	35.9 (8.97)
BMI [kg/m2], mean (sd)	18.79 (4.12)
Comorbidities:	
- epilepsy	3
- insulin dependent diabetes	1
- visual disorder corrected with glasses	5
- auditory limitations	1

*sd* standard deviation, *BMI* Body Mass Index

### Study protocol

The study protocol this prospective observational study was approved by the local Bioethics Commission of the Medical Faculty (5/2/2017) and was registered with Australian New Zealand Clinical Trials Registry (ACTRN12617000436370). Experimental conditions conformed to the Declaration of Helsinki.

### Procedure and measures

At the first stage original version of WGS was used to assess gait in the patients with hemiplegic cerebral palsy. The WGS, consisting of four subscales, evaluates 14 gait parameters which can be observed in the affected leg during consecutive gait stages, i.e. stance, toe off, swing and heel strike phases. Additionally, it accounts for the use of hand held gait aid while walking. The first subscale is designed to assess spatiotemporal gait parameters, while kinematic parameters are evaluated by subscale one, two, three and four. In all the items of the scale subjects can score from 1 to 3 points, except for Item One (1–5 points) and Item Eleven (1–4 points). The total number of points falls between 13.35 and 42, a higher score corresponding to greater gait impairments.

WGS assessment was performed based on video material acquired during trails registered with synchronised system designed for three-dimensional recording (BTS Smart system). For this purpose, two video cameras were located at two different places and simultaneously recorded images in the frontal and sagittal plane. The camera recording the frontal plane view was set in the middle of the delineated route, at a distance of two metres from the path walked by the subject. The camera recording the sagittal plane view was placed in line with the path walked. In the case of each subject, six trials comprising at least three complete gait cycles were recorded. Ultimately, the video material used by the rater for gait assessment provided back and front as well as left and right side view of the patient. The subjects were asked to walk at a comfortable, self-selected speed, and they were allowed to use their own orthopaedic aids.

The video material was analysed and the WGS based gait assessment was performed independently by three physical therapists with expertise in gait disorders associated with hemiplegic cerebral palsy, and familiar with assessment criteria used in WGS. While assessing the video recordings the three physiotherapists were unable to perform complete assessment with the original version of WGS in all the children, and to determine the final score, because in two points of WGS (item 4 - weight shift to the affected side and item 11 - knee flexion from toe off to mid swing) the gait patterns did not match any description. Complete gait assessment could not be performed in 16 out of the 34 children in the study group. More specifically in item 4 of WGS some subjects presented with decreased shift or very limited shift but not over the affected foot but over the unaffected foot, because head and trunk for part of the duration of the stance phase or for the entire duration of the stance phase were leaning towards the affected side. Assessment in item 11 of WGS was impossible due to the fact that some patients were found with increased unaffected knee flexion or maximal flexion in affected knee rather than with decreased or minimal flexion in affected knee.

Due to the fact that in the first phase it was impossible to perform complete assessment of gait pattern with WGS, including items 4 and 11, each of these points was discussed in detail and then points 4 and 11 were expanded and a common opinion was specified with regard to the gait patterns observed in the subjects. At the second stage of the study a modified WGS was introduced and the same video recordings were rescored by the same three physiotherapists, after 2 weeks, using the new, modified paediatric version of WGS (Table 2).

**Table 2:** Comparison of the original and modified Wisconsin Gait Scale in items 4 and 11

Original Wisconsin Gate Scale	Modified Wisconsin Gait Scale
4. Weight Shift to the Affected Side, with or without a gait aid	4. Weight Shift to the weight bearing leg, with or without a gait aid
1 = Full shift	1 = Full shift
2 = Decreased shift: head and trunk crosses midline, but not over the affected foot	2a = Decreased shift: head and trunk crosses midline, but not over the affected foot
3 = Very limited shift: head and trunk does not cross midline, minimal weight shift in the direction of the affected side	2b = Decreased shift: head and trunk crosses midline, but not over the unaffected foot, head and trunk for part of stance phase leaning towards the affected side
	3a = Very limited shift: head and trunk does not cross midline, minimal weight shift in the direction of the affected side
	3b = Very limited shift: head and trunk does not cross midline, minimal weight shift in the direction of the unaffected side, head and trunk during entire stance phase leaning towards the affected side
11. Knee flexion from toe off to mid swing	11. Knee flexion from toe off to mid swing
1 = normal (affected knee flexes equally to unaffected side)	1 = normal (affected knee flexes equally to unaffected side)
2 = some (affected knee flexes, but less than unaffected knee)	2a = some (affected knee flexes, but less than unaffected knee)
3 = minimal (minimal flexion noted in affected knee (hardly visible)	2b = some (affected knee flexes, but more than unaffected knee)
	3a = minimal (minimal flexion noted in affected knee (hardly visible)
	3b = maximal (maximal flexion noted in affected knee (well visible)
4 = none (knee remains in extension throughout swing)	4 = none (knee remains in extension throughout swing)

Inter-observer reliability of the modified WGS in the assessment of children with hemiplegic cerebral palsy was determined by comparing evaluation results acquired by three examiners independently analysing video recordings. Intra-observer reliability of the modified WGS in the assessment of children with hemiplegic cerebral palsy was determined by comparing evaluation results acquired by three examiners during two assessments carried out by each of them 2 weeks apart (test-retest).

### Statistical analysis

The scores were subjected to statistical analyses performed using Statistica 10.0 (StatSoft, Poland). Wilcoxon test was applied to assess test-retest differences independently for each of the physiotherapists as well as the relevant differences between the specific physiotherapists. Significance of correlations between the results was examined with Spearman’s correlation coefficient. Correspondence of test-retest results, for each of the physiotherapists and between the specific physiotherapists, was assessed with intra-class correlation coefficient (ICC) and value of intra-subject coefficient of variation (CV), which is calculated as a quotient of standard deviation and mean value in both measurements and shows relative variation between results obtained in both examinations. In order to determine what difference in two WGS-based measurements could be considered non-accidental, the minimal detectable change (MDC) was calculated. Repeatability of the results was calculated using Bland- Altman method. Statistical significance was assumed for *p* < 0.05.

### Sample size

The minimum size of the sample was calculated taking into account the number of children with spastic hemiplegic cerebral palsy treated at the rehabilitation clinic at Regional Hospital No. 2 in Rzeszów in 2014–2016. A fraction size of 0.8 was used, with a maximum error of 5%, a sample size of 30 patients was obtained. The study involved 34 children.

## Results

### General results

WGS score was determined for each patient six times, i.e. twice by three different physiotherapists. The following table presents the basic descriptive statistics characterizing WGS distribution in the specific series of measurement. The mean level of WGS score in the specific measurement series was very similar – on average differences between them were not higher than 0.5 point. There was also similar level of variation (standard deviation) - Table 3.

**Table 3:** Distribution of WGS in the specific measurement series

WGS	$\overline{x}$	Me	*sd*	min	max	95% c.i.
Physiotherapist 1 / exam 1	19.58	19.10	3.24	15.35	25.10	(18.45; 20.71)
Physiotherapist 1 / exam 2	19.64	19.10	3.05	15.35	25.10	(18.57; 20.70)
Physiotherapist 2 / exam 1	19.46	19.10	3.17	14.35	25.10	(18.35; 20.56)
Physiotherapist 2 / exam 2	19.75	19.60	3.10	15.10	25.10	(18.67; 20.83)
Physiotherapist 3 / exam 1	19.69	19.10	3.18	14.35	26.10	(18.58; 20.80)
Physiotherapist 3 / exam 2	19.86	20.23	3.26	14.35	26.10	(18.73; 21.00)

$\overline{x}$ – arithmetic mean, *Me* median, *sd* standard deviation, *min* minimum, *max* maximum, 95% c.i. – estimation of mean value in the entire population constructed as 95% confidence intervals

### Analysis of test vs. re-test

Comparison of results obtained using test-retest method showed no systematically oriented changes between the results determined during the two exams by any of the physiotherapists. Therefore, there are no grounds for claiming that the first examination produced higher or lower results than the second examination. Very low value of standard deviation in the differences between the two exams (for the specific physiotherapists amounting to 0.60; 0.72 and 0.94, respectively) allows a conclusion that deviations between the test-retest results do not exceed a few percent in relation to the outcome value (on average amounting to approx. 19.5 points) – Table 4.

**Table 4:** Comparison of test-retest results determined independently for each physiotherapist

	$\overline{x}$	Me	*sd*	min	max	95% c.i.
WGS (Physiotherapist 1)						
test	19.58	19.10	3.24	15.35	25.10	(18.45; 20.71)
re-test	19.64	19.10	3.05	15.35	25.10	(18.57; 20.70)
re-test vs. test (*p* = 0.4413)	0.06	0.00	0.60	− 1.00	2.00	(− 0.15; 0.27)
WGS (Physiotherapist 2)						
test	19.46	19.10	3.17	14.35	25.10	(18.35; 20.56)
re-test	19.75	19.60	3.10	15.10	25.10	(18.67; 20.83)
re-test vs. test (*p* = 0.0597)	0.29	0.00	0.72	−1.00	2.00	(0.04; 0.54)
WGS (Physiotherapist 3)						
test	19.69	19.10	3.18	14.35	26.10	(18.58; 20.80)
re-test	19.86	20.23	3.26	14.35	26.10	(18.73; 21.00)
re-test vs. test (*p* = 0.3109)	0.18	0.00	0.94	−2.00	3.00	(−0.15; 0.50)

$\overline{x}$ – arithmetic mean, *Me* median, *sd* standard deviation, *min* minimum, *max* maximum, 5% c.i. – estimation of mean value in the entire population constructed as 95% confidence intervals, *p* – Wilcoxon test probability values

**Fig. 2:**
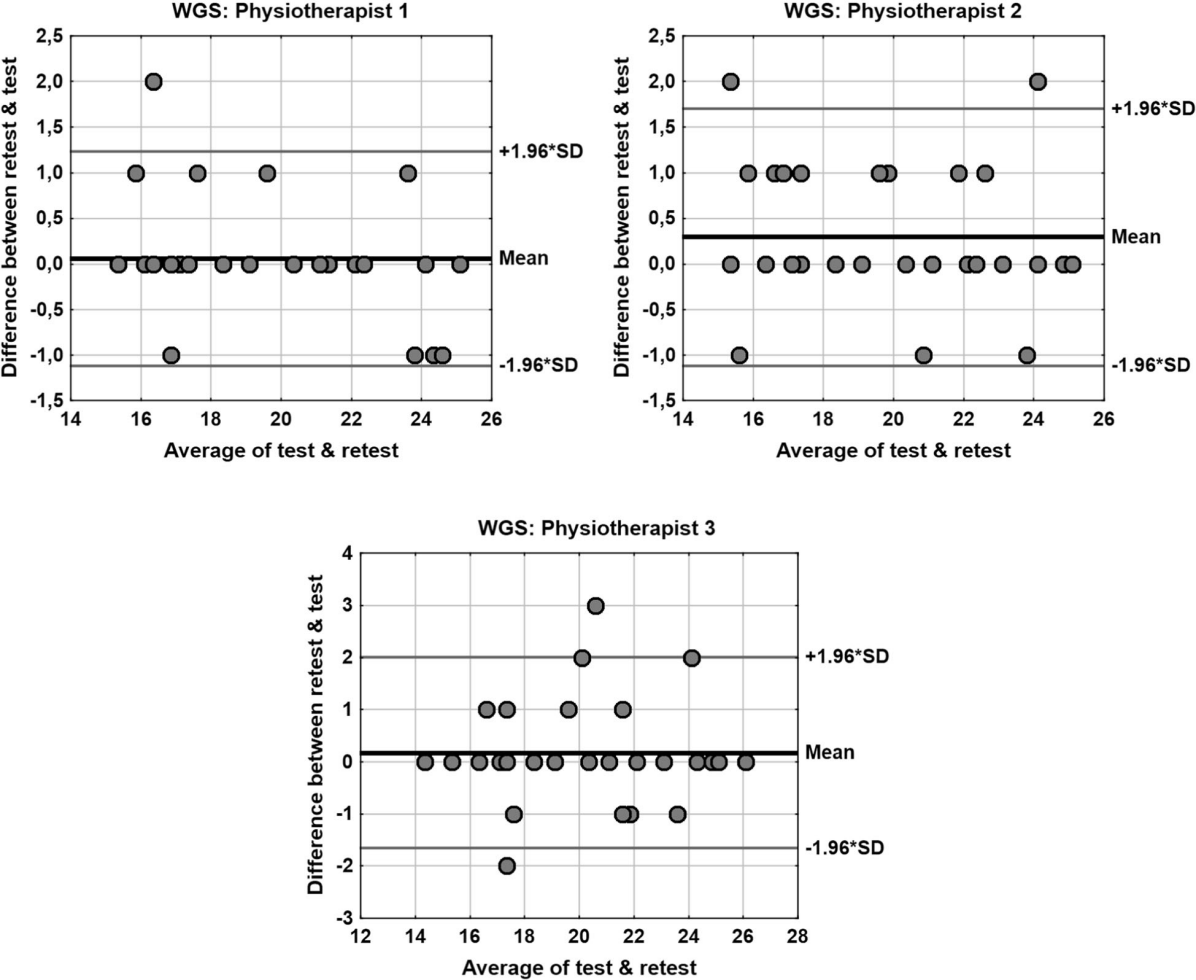
The Bland- Altman plots for comparison of test-retest results, separately for each physiotherapist

Findings of comparative analysis of the test-retest results are also shown in Table 5, which presents the result of Wilcoxon test, Spearman’s rank correlation coefficient with assessment of significance, intra-class correlation coefficient (ICC), and value of intra-subject coefficient of variation (CV) and minimal detectable change (MDC), between the two examinations (test-retest). All the figures show very good test-retest reliability. The findings show no systematically oriented differences between the two examination (insignificant value of Wilcoxon test), very high correlation between the scores (value of Spearman’s rank correlation coefficient 0.9 ≤ |*R*| < 1), very high ICC, low value of CV (up to 2.5% for the specific physiotherapists) and value of MDC up to 2 points. The Bland- Altman plots for comparison of test-retest results, separately for each physiotherapist are shown in Fig. 2.

**Table 5:** Comparison of test-retest results, separately for each physiotherapist

Physiotherapist	Comparison of test-retest				
	Wilcoxon test	Rank correlation	ICC	CV	MDC
1	0.4413	0.97 (*p* < 0.001)	0.9821	1.6%	1.20
2	0.0597	0.97 (*p* < 0.001)	0.9701	2.1%	1.52
3	0.3109	0.95 (*p* < 0.001)	0.9575	2.3%	1.82

*p* – test probability values, *ICC* intraclass correlation coefficient, *CV* intra-subject coefficient of variation, *MDC* minimal detectable change (calculated for 95% confidence level)

### Comparison of assessments made by the physiotherapists during the test and the retest

Analysis of consistency between scores determined by the specific physiotherapists during exam 1 (test) and exam 2 (retest) showed no systematically oriented differences between WGS values assigned to the patients by various physiotherapists; *p*-values calculated with Wilcoxon test significantly exceed 0.05 (Table 6).

**Table 6:** Paired comparison of the scores determined by the specific physiotherapists in exam 1 (test) and exam 2 (retest)

	$\overline{x}$	Me	*sd*	min	max	95% c.i.
WGS (total) exam 1 (test)						
Physiotherapist 2 vs. Physiotherapist 1 (*p* = 0.4446)	−0.12	0.00	0.81	−2.00	2.00	(−0.40; 0.16)
Physiotherapist 3 vs. Physiotherapist 2 (*p* = 0.2575)	0.23	0.00	1.15	−3.00	3.00	(−0.17; 0.63)
Physiotherapist 3 vs. Physiotherapist 1 (*p* = 0.3078)	0.11	0.00	0.58	−1.00	1.00	(−0.09; 0.31)
WGS (total) exam 2 (retest)						
Physiotherapist 2 vs. Physiotherapist 1 (*p* = 0.6529)	0.12	0.00	0.91	−1.00	3.00	(−0.20; 0.44)
Physiotherapist 3 vs. Physiotherapist 2 (*p* = 0.6292)	0.11	0.00	1.32	−4.00	2.00	(−0.35; 0.57)
Physiotherapist 3 vs. Physiotherapist 1 (*p* = 0.1702)	0.23	0.00	1.08	−3.00	2.00	(−0.15; 0.60)

$\overline{x}$ – arithmetic mean, *Me* median, *sd* standard deviation, *min* minimum, *max* maximum, 5% c.i*.* – estimation of mean value in the entire population constructed as 95% confidence intervals, *p* – Wilcoxon test probability values

**Fig. 3:**
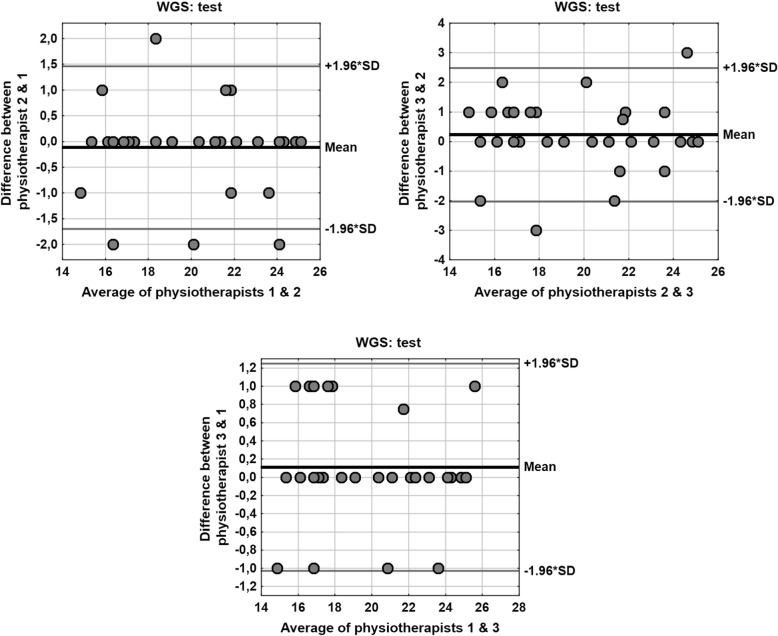
The Bland- Altman plots for paired comparison of the scores between the specific physiotherapists in exam 1 (test)

**Fig. 4:**
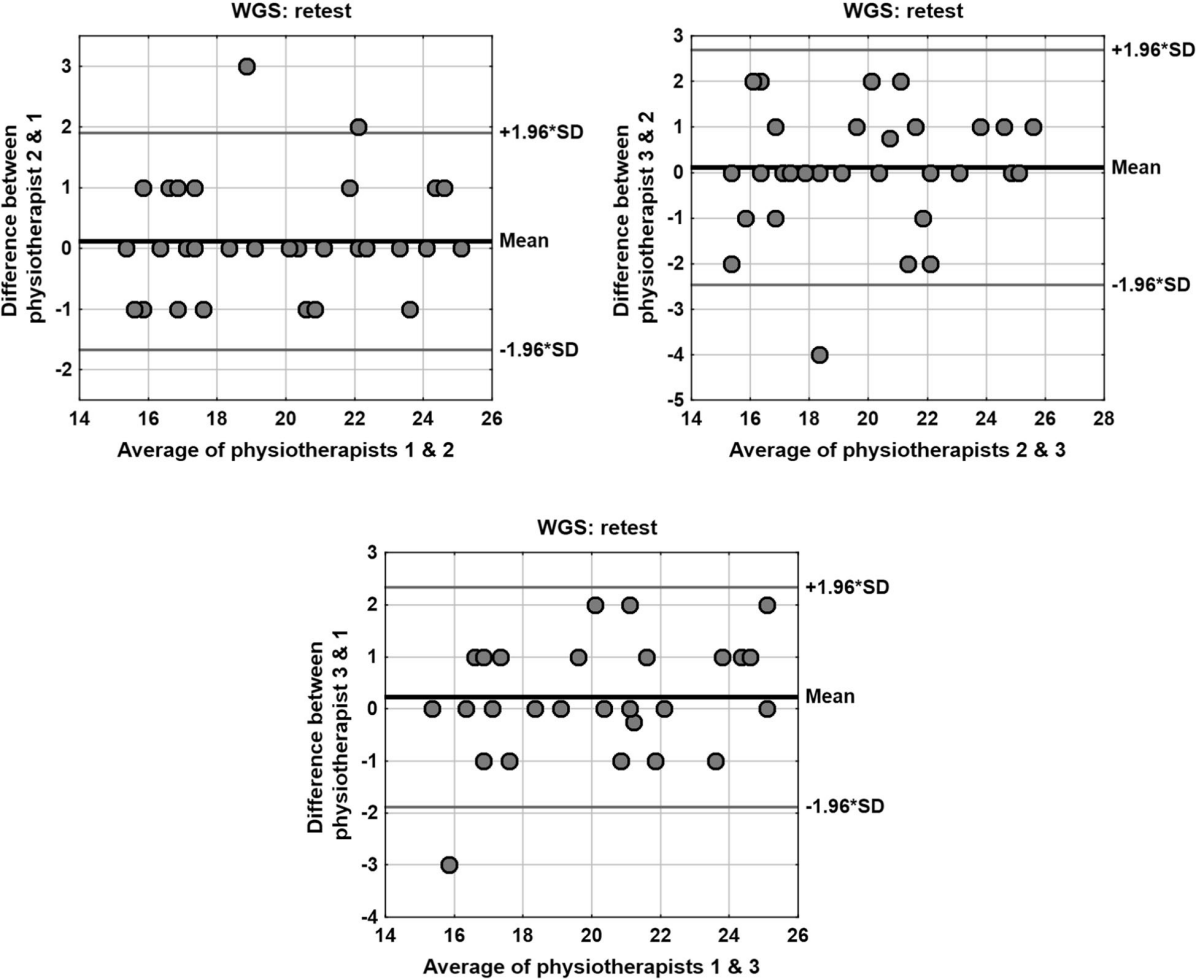
The Bland- Altman plots for paired comparison of the scores between the specific physiotherapists in exam 2 (retest)

Another important issue is the fact that correlations between assessments performed by the physiotherapists in exam 1 (test) and exam 2 (retest) were very high (value of Spearman’s rank correlation coefficient 0.9 ≤ |*R*| < 1); only in exam 2 (retest) the correlation Physiotherapist 3 vs. Physiotherapist 2 was 0.7 < |R| < 0.9. A wider range of statistics related to the paired comparison of assessments performed by the specific physiotherapists is presented in Table 7. The values of all the defined measures and coefficients show very high consistency of the results determined by the physiotherapists. The Bland- Altman plots for paired comparison of the scores between the specific physiotherapists in exam 1 (test) and in exam 2 (retest) are shown in Figs. 3 and 4.

**Table 7:** Paired comparison of the scores between the specific physiotherapists in exam 1 (test) and in exam 2 (retest)

	Wilcoxon test	Rank correlation	ICC	CV	MDC
Physiotherapist	Exam 1 (test)				
2 vs. 1	0.4446	0.96 (*p* < 0.001)	0.9685	2.1%	1.59
3 vs. 2	0.2575	0.92 (*p* < 0.001)	0.9335	3.1%	2.26
3 vs. 1	0.3078	0.98 (*p* < 0.001)	0.9835	1.6%	1.15
Physiotherapist	Exam 2 (re-test)				
2 vs. 1	0.6529	0.94 (*p* < 0.001)	0.9564	2.4%	1.77
3 vs. 2	0.6292	0.88 (*p* < 0.001)	0.9162	3.5%	2.49
3 vs. 1	0.1702	0.91 (*p* < 0.001)	0.9410	2.9%	2.05

*p* – test probability values, *ICC* intraclass correlation coefficient, *CV* intra-subject coefficient of variation, *MDC* minimal detectable change (calculated for 95% confidence level)

## Discussion

Researchers have been looking for an optimal tool designed for systematic assessment of gait in children with spastic hemiplegic cerebral palsy. The inspiration for this study was the fact that whereas classifications taking into account community involvement, activity, hand function as well as secondary conditions in children with cerebral palsy are widely available in the literature [22–27], there are few scales focused on assessment of the walking pattern in this group of patients [7, 10–13]. Furthermore, there is no specific scale enabling multivariate assessment of both spatiotemporal and kinematic gait parameters designed typically for children with spastic hemiplegic cerebral palsy.

Observation gait scales are an auxiliary tool in the gait analysis of children over 6 years of age, allowing for a basic assessment of the gait pattern [11]. The scales available for assessing walking skills in children with cerebral palsy focus only of examining kinematic gait parameters [10–13]. On the other hand, WGS is a simple, ordinal scale based on observation. The scale does not measure specific spatiotemporal and kinematic parameters, yet it enables a subjective assessment and categorisation of gait patterns into orderly groups, providing however only global description of gait. Thus the scale describes positions of parts of the lower limbs and joints in the gait cycle of the affected and unaffected legs. Descriptions of the walking pattern refer mainly to the symmetry of the gait. The scale is divided into subscales which may correspond to temporal (stance time), spatial (step length, stance width) and kinematic parameters of hip, knee, ankle and pelvis joints, in the sagittal, transverse, and frontal planes [14–18, 21].

The present study is part of a larger research project where the authors have performed detailed assessment of test-retest reliability and internal consistency of WGS [28], and have examined 3-diemensional gait parameters in relation to WGS-based observational gait assessment in patients with post-stroke hemiparesis [15]. The above studies demonstrated that, in addition to being an easy-to-use tool, WGS can effectively assess walking ability in hemiparetic patients after stroke, and it is characterised by high internal consistency and test-retest reliability. Ultimately, it was also shown that there was a moderate and good level of correspondence between spatiotemporal parameters identified during 3-dimensional gait examination and results of gait assessment based on observational WGS [15, 28]. The acquired results have encouraged the authors to carry out further research to investigate feasibility of WGS based assessment in other groups of neurological patients with hemiplegia. Furthermore, Gor-García-Fogeda and co-authors emphasize the importance of this type of research and recommend more in-depth analysis of psychometric properties of observational gait scales, including WGS, in patients with varied neurological disorders other than stroke [21]. In view of the above, the present study is the first report from research designed as an attempt to adapt WGS scale for children with spastic hemiplegic cerebral palsy.

The present findings show very good intra-observer reliability of the modified WGS (consistency of test-retest results independently for each physiotherapist). This was reflected by a lack of systematically oriented differences between the test-retest measurements (insignificant result in Wilcoxon test), very high value of Spearman’s rank correlation coefficient 0.9 ≤ |*R*| < 1, very high value of ICC > 0.9, and low value of CV < 2.5% for the specific physical therapists. It was also shown there was very good inter-observer reliability of the modified WGS (consistency of results between the specific physiotherapists in the first exam and in the second exam). This was also reflected by a lack of systematically oriented differences between WGS scores assigned to the patients by the different physiotherapists (insignificant result in Wilcoxon test), very high value of Spearman’s rank correlation coefficient. Furthermore, the determined values of ICC and CV also reflect very high consistency of the results between the physiotherapists.

Evaluation of intra and inter-rater reliability has been in focus of numerous studies related to available scales enabling assessment of gait in children with cerebral palsy. For example, Araújo and co-authors examined intra- and inter-rater reliability of the Observational Gait Scale (OGS) for children with spastic cerebral palsy. In accordance with the study design, the OGS was applied in the process of rating 23 videos of children with spastic diplegia and hemiplegic cerebral palsy. The assessment was performed in two sessions, by four physical therapists, who had been trained on the use of the OGS and instructed about the significance of all the items of the scale. In order to avoid memory bias the second evaluation was performed 2 weeks after the first one. Each rater was provided with a CD containing the OGS file as well as video material presenting frontal and sagittal plane view of each subject examined. The authors established that the OGS presented very good intra-rater reliability for the hip (*r* = 0.73), knee (*r* = 0.77) and ankle/foot complex (*r* = 0.79), and good reliability for the pelvis (*r* = 0.59). Very good inter-rater reliability was identified for the knee (*r* = 0.65), and ankle/foot complex (*r* = 0.68), while good reliability was shown for the hip (*r* = 0.48). All of the above relationships were statistically significant [29]. Similar issues were investigated by Dickens and Smith who evaluated reliability of a visual assessment of gait based on the Physician Rating Scale in children with hemiplegic cerebral palsy. Evaluation of the Visual Gait Assessment Scale (VGAS), in this case performed by two expert raters, was based on video material showing 31 hemiplegic children, ranging in age from 5 to 17 years. The version used in the study was developed with the aim to evaluate the position of hip, knee, ankle and foot in the sagittal plane. The highest intra-rater reliability was demonstrated in the case of initial contact and foot contact during the stance phase. On the other hand, better inter-rater reliability was reported for foot contact during stance and heel-off during the terminal stance. Conversely, poor reliability was found for hip parameters, particularly in the swing phase [11]. Likewise, Brown and colleagues evaluated reliability of the VGAS for children with hemiplegic cerebral palsy when used by experienced and inexperienced observers. Four experienced and six inexperienced observers viewed videotaped footage of four children with hemiplegic cerebral palsy on two separate occasions. The experienced observers generally had higher inter-observer and intra-observer reliability than the inexperienced observers. Both groups showed higher agreement for assessments made at the ankle and foot than at the knee and hip. The authors argue that VGAS can be used by inexperienced observers but is limited to observations in the sagittal plane and by poor reliability at the knee and hip for experienced and inexperienced observers [30].

The present findings suggest that WGS, originally designed for gait assessment in adults after stroke, can in fact be successfully used in children with spastic hemiplegic cerebral palsy. This provides encouragement for the authors to carry out further research focused on detailed analysis of psychometric properties of the new, paediatric version of WGS applied in this group of patients.

## Conclusion

The findings show very good intra- and inter-observer reliability of the modified WGS. The new, ordinal, paediatric version of WGS, proposed by the authors, seems to be useful as an additional tool that can be used in qualitative observational gait assessment of children with spastic hemiplegic cerebral palsy. Practical dimension of the study lies in the fact that it proposes a simple, easy-to-use tool for a global gait assessment in children with spastic hemiplegic cerebral palsy. However, further research is needed to validate the modified WGS by comparing it to other observational scales and objective 3-dimensional spatiotemporal and kinematic gait parameters.

## Abbreviations

CV: Intra-subject coefficient of variation
ICC: Intra-class correlation coefficient
WGS: Wisconsin Gait Scale
